# Enhancement of the CXCL12/CXCR4 axis due to acquisition of gemcitabine resistance in pancreatic cancer: effect of CXCR4 antagonists

**DOI:** 10.1186/s12885-016-2340-z

**Published:** 2016-05-12

**Authors:** Mamoru Morimoto, Yoichi Matsuo, Shuji Koide, Ken Tsuboi, Tomoya Shamoto, Takafumi Sato, Kenta Saito, Hiroki Takahashi, Hiromitsu Takeyama

**Affiliations:** Department of Gastroenterological Surgery, Nagoya City University Graduate School of Medical Science, Kawasumi 1, Mizuho-cho, Mizuhoku, Nagoya 467-8601 Japan

**Keywords:** Pancreatic cancer, Gemcitabine resistant, CXCR4, CXCL12, CXCR4 antagonist

## Abstract

**Background:**

The CXCL12-CXCR4 signaling axis in malignant tumor biology has increased in importance, and these peptides are implicated in tumor growth, invasion and metastasis. The aim of our study was to examine the important role of the axis in pancreatic cancer (PaCa) cells’ relationship with stromal cells in gemcitabine-resistant (GEM-R) tumors and to confirm the effectiveness of CXCR4 antagonists for the treatment of GEM-R PaCa cells.

**Methods:**

We established two GEM-R PaCa cell lines using MIA PaCa-2 and AsPC-1 cells. The expression of *CXCR4* mRNA in PaCa cells and the expression of *CXCL12* mRNA in fibroblasts were examined by reverse transcription polymerase chain reaction (RT-PCR). The expression of CXCR4 protein in PaCa cells was examined by immunosorbent assay (ELISA) and immunocytochemistry. Using Matrigel invasion assays and animal studies, we then examined the effects of two CXCR4 antagonists, AMD11070 and KRH3955, on the invasiveness and tumorigenicity of GEM-R PaCa cells stimulated by CXCL12.

**Results:**

We found that the expression of CXCR4 in GEM-R PaCa cells was significantly enhanced by GEM but not in normal GEM-sensitive (GEM-S) PaCa cells. In RT-PCR and ELISA assays, the production and secretion of CXCL12 from fibroblasts was significantly enhanced by co-culturing with GEM-R PaCa cells treated with GEM. In Matrigel invasion assays, the invasiveness of GEM-R PaCa cells treated with GEM was significantly activated by fibroblast-derived CXCL12 and was significantly inhibited by CXCR4 antagonists, AMD11070 and KRH3955. *In vivo*, the tumorigenicity of GEM-R PaCa cells was activated by GEM, and it was significantly inhibited by the addition with CXCR4 antagonists.

**Conclusions:**

Our findings demonstrate that the CXCL12-CXCR4 signaling axis plays an important role in PaCa cells’ resistance to GEM. CXCR4 expression was significantly enhanced by the exposure to GEM in GEM-R PaCa cells but not in GEM-S PaCa cells. Furthermore, CXCR4 antagonists can inhibit the growth and invasion of GEM-R PaCa cells. These agents may be useful as second-line chemotherapy for GEM-R PaCa in the future.

**Electronic supplementary material:**

The online version of this article (doi:10.1186/s12885-016-2340-z) contains supplementary material, which is available to authorized users.

## Background

Pancreatic cancer (PaCa) has the worst survival rate of all cancers. PaCa remains the fourth leading cause of cancer death in the United States [1]. With surgery, radiotherapy and chemotherapy, the 5-year survival rate of patients with PaCa remains less than 5 % [2]. There has been some progress in the use of improved diagnostic methods and development of novel targeted therapies. Gemcitabine (GEM) was approved in 1997 as a first-line chemotherapeutic drug for patients with locally advanced or metastatic PaCa [3]. GEM remains the standard treatment for pancreatic cancer patients. However, it has not proven very effective clinically, and improvement in a patient’s survival undergoing GEM therapy is minimal [4]. Clinical experience has shown that there is a transient effect of GEM therapy on PaCa after beginning chemotherapy; however, resistance to GEM readily appears.

Recently, the role of chemokines in malignant tumor biology has increased in importance because studies have shown that these peptides may influence tumor growth, invasion, and metastasis [5–14]. We have reported that two chemokines, CXCL8 and CXCL12, play important roles in the angiogenesis of PaCa [5]. The functional roles of CXCR4 in cell migration [14, 15] and cell proliferation [16] in response to CXCL12 have been suggested in malignant diseases. Furthermore, activation of the CXCL12-CXCR4 signaling axis is involved in conferring chemoresistance to PaCa cells through potentiation of intrinsic survival mechanisms [17].

CXCR4 antagonists were initially developed as new drugs for the treatment of HIV-1 infection [18–20]. With the rapid increase in our knowledge of non-HIV-related functions of CXCR4, other potential applications for treatment of cancer have emerged and have gradually replaced the original intent to use CXCR4 antagonists as anti-HIV drugs. There have been several reports describing the effects of CXCR4 antagonists (AMD3100, AMD11070 and KRH3955) in the treatment of malignant tumors, including breast cancer [21], small cell lung cancer [22], cholangiocarcinoma [23], gastric cancer [24] and pancreatic cancer [17, 25, 26]. However, it has not been reported whether the activated CXCL12–CXCR4 signaling axis plays an important role in PaCa cells’ resistance to GEM and whether CXCR4 antagonists can inhibit the activated signaling axis in GEM-resistant (GEM-R) PaCa *in vitro* and *in vivo*.

The purpose of this study was to determine the roles of the CXCL12–CXCR4 signaling axis in the relationship between tumor and stromal cells in GEM-R PaCa. Furthermore, we examined the therapeutic significance of CXCR4 antagonists, AMD11070 and KRH3955, in preventing the rescue effect of activated CXCL12-CXCR4 signaling. This is the first report that reveals the important role of the CXCL12-CXCR4 signaling axis in enhancing resistance to GEM and the effect of CXCR4 antagonists on GEM-resistant PaCa cells.

## Methods

### Cell lines and culture conditions

Human pancreatic cancer (PaCa) cell lines MIA PaCa-2 and AsPC-1 and human dermal fibroblast were obtained from the American Type Culture Collection (Rockville, MD) and Kurabo Industries (Osaka, Japan), respectively. The cell lines were maintained at 37 °C with 5 % CO_2_ in a humidified atmosphere. The following media were used: (1) MIA PaCa-2 cells were cultured in Dulbecco’s Modified Eagle’s Medium (DMEM), (2) AsPC-1 cells were incubated in Roswell Park Memorial Institute (RPMI-1640) medium (Sigma Aldrich, St Louis, MO, USA) supplemented with 10 % fetal bovine serum (FBS) and antibiotics and (3) fibroblasts (FB) were maintained in fibroLife S2 Comp kit (Kurabo Industries Ltd., Osaka, Japan) supplemented with 2 % FBS.

### Establishment of PaCa cell lines resistant to gemcitabine

Gemcitabine (GEM) was purchased from Toronto Research Chemicals, Inc. (Toronto, Ontario, Canada). First, we determined the half maximal inhibitory concentration (IC50) of GEM for MIA PaCa-2 or AsPC-1 cells using the Premix WST-1 Cell Proliferation Assay System (Takara Bio, Japan) according to the manufacturer’s instruction. Briefly, MIA PaCa-2 or AsPC-1 cells were seeded at a density of 2 × 10^3^ cells per 100 μL in 96-well plates and allowed to adhere overnight. Then, cultures were re-fed with fresh media containing various concentrations of GEM. After 72 h of incubation, absorbance was measured at 450 nm in each well using a SpectraMax 340 spectrophotometer (Molecular Devices, CA, USA). The IC50 of GEM for each pancreatic cancer line was determined by constructing a dose-response curve. Each pancreatic cancer cell line was passaged in the cell lines’ IC50 concentration of GEM for 2 to 3 weeks. After passage, we again determined the cell lines’ IC50 value for GEM. Then, each pancreatic cancer cell line was passaged in the cell lines’ re-determined IC50 concentration of GEM for 2 to 3 weeks. The process was repeated at increasing doses of GEM until the cell lines demonstrated at least a 50-fold greater IC50 value for GEM than the parental cell lines. The resultant cell lines were resistant to GEM at a concentration of 20 μM.

### Proliferation assay

The proliferation assay was conducted using the Premix WST-1 Cell Proliferation Assay System (Takara Bio, Japan) according to the manufacturer’s instruction. Briefly, GEM-resistant (GEM-R) and GEM-sensitive (GEM-S) MIA PaCa-2 or AsPC-1 cells were seeded at a density of 2 × 10^3^ cells per 100 μL in 96-well plates and allowed to adhere overnight. Then, cultures were re-fed with fresh media containing various concentrations (0–100 μM) of GEM. After 72 h of incubation, absorbance was measured at 450 nm in each well using a SpectraMax 340 spectrophotometer.

### Enzyme-linked immunosorbent assays (ELISAs)

The expression of CXCR4 protein by GEM-R/S MIA PaCa-2 and AsPC-1 cells was examined using the CXCR4 ELISA kit (USCN Life Science Inc., Wuhan, China) according to the manufacturer’s instructions. A total of 1 × 10^5^ GEM-R/S cells were seeded in each 100 mm dish. Then, we added different concentrations of GEM (0 – 20 μM), and the cells were incubated for 72 h. After indicated treatments, cell lysates were prepared. A total of 150 μg of protein was taken for ELISA assay. Similarly, CXCL12 levels in the supernatant from FB co-cultured with GEM-R/S MIA PaCa-2 cells were determined using the CXCL12 ELISA kit (R&D, Minneapolis, MN, USA) according to the manufacturer’s instruction. To determine the synergistic effect of the tumor-stromal interaction, we cultured FB (1.0 × 10^6^ cells in 6-well plates) with or without GEM-R/S [1.0 × 10^6^ cells on inserts with 0.4-μm pores (Thermo Scientific, Rockford, IL, USA)] for 72 h using a double chamber method. After the incubation, the media were collected and microfuged at 1500 rpm for 5 min to remove particles. The supernatants were frozen at -80 °C until use. A total of 150 μg of the protein was taken for ELISA assay.

### RNA isolation and reverse transcription polymerase chain reaction (RT-PCR)

Total RNA was extracted from cell pellets using an RNeasy Plus Mini Kit (Qiagen, TX, USA), and RT-PCR was performed using Superscript III First-strand Synthesis SuperMix for qRT-PCR (Invitrogen, Carlsbad, CA, USA). The concentration of each cDNA was measured with a NanoDrop1000 (Thermo Fisher Scientific, DE, USA) and adjusted to 40 ng/mL with diethylpyrocarbonate (DPEC)- treated water. We performed real-time PCR with FAM-labeled TaqMan probes (*CXCR4*: Hs00607978_s1; *CXCL12*: Hs03676656_mH; *GAPDH*: Hs99999905_m1; CXCR7: Hs00664172_s1 (Applied Biosystems, Foster City, CA, USA)) and TaqMan Universal Master Mix (Applied Biosystems) using Chromo4 (BioRad, MA, USA). PCR was carried out by an initial incubation at 50 °C for 2 min, followed by denaturation at 95 °C for 10 min and 50 cycles of 95 °C for 15 s and 60 °C for 1 min.

### Immunocytochemical staining

The expression of CXCR4 protein in GEM-R/S MIA PaCa-2 cells was detected by immunostaining. Three days after treating with GEM, GEM-R/S PaCa cells were washed twice with ice-cold PBS, fixed in 4 % paraformaldehyde for 20 min at room temperature and washed twice with ice-cold PBS. The cells were then incubated for 15 min in PBS containing 0.5 % Triton X-100, washed with PBS, blocked in 1 % BSA in PBS for 30 min and incubated with rabbit anti-CXCR4 polyclonal antibody (1:100, Abcam, Cambridge, UK) at 4 °C overnight. Subsequently, the cells were washed with PBS, incubated with Alexa Fluor 488 goat anti-rabbit IgG (H + L) (1:100, Life Technologies, Carlsbad, CA) and mounted with Prolong® Gold Antifade Reagent with DAPI (Life Technologies, Carlsbad, CA).

### Invasion assay

*In vitro* invasion assays were performed using the BD Bio-Coat Matrigel invasion assay system (BD Biosciences, Franklin Lakes, NJ) according to the manufacturer’s instructions. Briefly, GEM-R/S cells (2.5 × 10^4^ cells) were seeded into the Matrigel precoated Transwell chambers consisting of polycarbonate membranes with 8.0 μm pores. The Transwell chambers were then placed into 6-well plates, into which we added basal medium only or basal medium containing various concentrations of recombinant CXCL12. After incubating GEM-R/S cells for 22 h, the upper surface of the Transwell chambers was wiped with a cotton swab and the invading cells were fixed and stained using Diff-Quick cell staining kit (Dade Behring, Inc., Newark, DE). The number of invading cells was counted in 5 random microscopic fields (200×). To confirm whether the invasive potency of PaCa cells was increased by FB-derived CXCL12 and inhibited by the CXCR4 antagonists, AMD11070 (AdooQ BioScience, Irvine, CA) and KRH3955 (Kureha Chemical Industry, Tokyo, Japan), we performed an invasion assay for GEM-R/S cells using a double-chamber method. Briefly, we co-cultured GEM-R/S cells (2.5 × 10^4^ cells in Transwell chambers) with FB (1 × 10^4^ cells in 6-well plates) blocking with or without CXCR4 antagonists, AMD11070 and KRH3955, at a concentration of 1 μM. After incubation for 22 h, invading cells were counted in the same manner.

### Animals

All animal studies were conducted in accordance with the guidelines established by the internal Institutional Animal Care and Use Committee and Ethics Committee guidelines of Nagoya City University.

Female BALB/c nu-nu mice (5 to 6 weeks old) were obtained from Charles River (Sulzbach, Germany). The animals were housed in standard Plexiglas cages (8 per cage) in a room maintained at constant temperature and humidity and in a 12 h/12 h light-dark cycle. Their diet consisted of regular autoclaved chow and water *ad libitum*. All animal experiments were approved by the authorities in our institute and were in compliance with the institution’s guidelines.

### Subcutaneous transplant animal model

A total of 2 × 10^7^ MIA PaCa-2 cells were injected subcutaneously into mice. Tumors were measured weekly and tumor volume was documented. Tumors were allowed to grow until they reached a volume of 1 cm^3^, at which time the mice were sacrificed and the tumor tissue was harvested. For serial transplantation, the harvested tumor tissues were chopped into pieces approximately 1 to 2 mm^3^ in dimension. Tumor pieces were implanted subcutaneously into the mice. GEM and CXCR4 antagonists were administered 3 weeks after tumor implantation as follows: 25 mg GEM/kg body weight, 1 mg AMD11070, and 1 mg KRH3955/kg body weight were given intraperitoneally every week.

### Experimental protocol

Mice were randomly assigned to 1 of the following 6 treatment groups (4 mice per group): group I was not given any drugs; group II was given GEM alone; group III was given AMD11070 alone; group IV was given KRH3955 alone; group V was given GEM plus AMD11070; group VI was given GEM plus KRH3955. Therapy was continued for 4 weeks, and the mice were sacrificed 2 weeks later. We calculated the tumor volume according to the following formula: tumor volume (mm^3^) = d^2^ x D/2, where d and D were the shortest and longest diameter, respectively. Finally, the tumors were harvested from mice after the treatment and fixed in formaldehyde for further analysis.

### Immunohistochemical analysis

Formalin-fixed, paraffin-embedded mouse tumor tissue sections were mounted on 3-amino-propyltriethoxylsilane-coated slides. Dewaxed paraffin sections were placed in a microwave (10 min, 600 watts) to recover antigens before staining. Antibodies used were as follows: rabbit anti-CXCR4 polyclonal antibody, rabbit anti-SDF-1α polyclonal antibody (1:50) and, rabbit anti-Hypoxia-Inducible Factor (HIF)-1α monoclonal antibody (1:100) (Abcam, Cambridge, UK), followed by secondary antibodies conjugated to biotin. Peroxidase-conjugated streptavidin was used with 3,3-diaminobenzidine tetrahydrochloride (DAB) (Biocare Medical, Concord, CA, USA) as the chromogen for detection. Hematoxylin was used for nuclear counterstaining. CXCR4-positive PaCa cells, CXCL12-positive stromal cells and HIF-1α-positive PaCa cells exhibited DAB-positive (brown) staining; negative cells were stained with the hematoxylin counterstain only. The number of CXCR4-immunoreactive cells in mouse specimens was expressed as a percentage of the total number of cells that were randomly counted in 10 fields at × 400 magnification. For each image, a color deconvolution method was used to isolate CXCL12-positive and HIF-1α-positive DAB-stained cells from CXCL12-negative and HIF-1α-negative hematoxylin-stained cells. DAB and hematoxylin were digitally separated using ImageJ software (version 1.46c; WS Rasband, National Institutes of Health, Bethesda, MD, USA, http://rsb.info.nih.gov/ij/) and an ImageJ plugin for color deconvolution that calculated the contribution of DAB and hematoxylin, based on stain-specific red-green-blue (RGB) absorption. Following deconvolution, the scale was set to the 200 μm scale bar on each image. The measurement parameter was integrated optical density (IOD). Optical density was calibrated and the area of interest was set as follows: hue, 0–30; saturation, 0–255; intensity, 0–255. Then, the values were counted. The IOD was log_10_ transformed [27].

### Nuclear factor-kappa B (NF-κB) activity

The activity of NF-κB was measured using NF-κB (p65) transcription factor assays.　A total of 1 × 10^5^ GEM-R/S cells of MIA PaCa-2 cells were seeded in 100-mm dishes and incubated with different concentrations of GEM for 72 h. After indicated treatments, nuclear proteins were extracted using NE-PER Nuclear and Cytoplasmic Extraction Reagents (Thermo Scientific, IL, USA). The concentrations of nuclear proteins were measured using a Pierce BCA Protein Assay Kit (Thermo Scientific), and protein concentrations were adjusted for equal loading (200 μg/mL). The levels of NF-κB p65 protein detected with the NF-κB p65 ELISA kit (Invitrogen, USA) according to the manufacturer’s instructions.

### Statistical analysis

All measurement data were expressed as means ± standard deviation (SD). They were calculated for experiments performed in triplicate (or more). Multiple group comparisons were performed by using one-way analysis of variance (ANOVA) followed by the Dunnett test, and Bonferroni tests were used for *post hoc* 2-sample comparisons. A two-sided *p*-value of less than 0.05 was considered statistically significant. All statistical analyses were performed using EZR (Saitama Medical Center, Jichi Medical University, http://www.jichi.ac.jp/saitama-sct/SaitamaHP.files/statmedEN.html; Kanda, 2012), a graphical user interface for R (The R Foundation for Statistical Computing, Vienna, Austria, version 2.13.0). More precisely, EZR is a modified version of R Commander (version 1.6-3) that was designed to add statistical functions frequently used in biostatistics.

## Results

### The effect of GEM on the proliferation of GEM-R PaCa cells *in vitro*

**Fig. 1:**
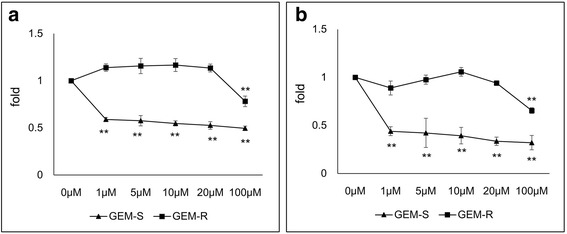
Effect of GEM on the proliferation of GEM-R and GEM-S PaCa cell lines. **a** The proliferation of GEM-R and GEM-S MIA PaCa-2 cells (**b**) and AsPC-1 cells was determined using WST-1 assays. Values are expressed as means ± SD. Statistical significance was analyzed by using one-way ANOVA followed by Dunnett’s test. **, *P* < 0.01; *, *P* < 0.05 versus the control (0 μM)

We first determined how GEM affected the proliferation of PaCa cells that were sensitive or resistant to the drug. We used 2 GEM-R PaCa cell lines, MIA PaCa-2 and AsPC-1. With these 2 cell lines, we found that GEM significantly inhibited GEM-S cell proliferation in a dose-dependent manner (*P* < 0.01); however, it could not inhibit GEM-R cell proliferation at the doses used (Fig. 1a, MIA PaCa-2; Fig. 1b, AsPC-1).

### The expression of CXCR4 in GEM-R PaCa cells was enhanced by GEM

**Fig. 2:**
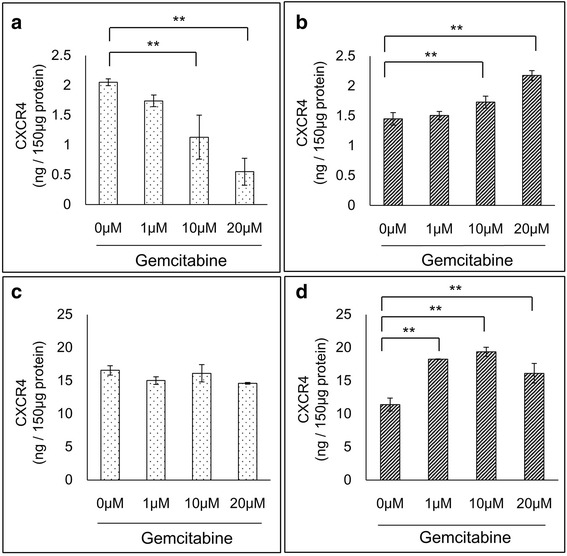
Alteration of CXCR4 protein expression in PaCa cells by GEM. PaCa cells were treated with different concentrations of GEM (0–20 μM) for 72 h. The concentrations of CXCR4 protein in **a** MIA PaCa-2 GEM-S, **b** MIA PaCa-2 GEM-R, **c** AsPC-1 GEM-S **d** and AsPC-1 GEM-R were measured by ELISA. Values are expressed as means ± SDs. Multiple comparisons were performed by using one-way ANOVA followed by Dunnett’s test. **, *P* < 0.01; *, *P* < 0.05 versus control (0 μM)

**Fig. 3:**
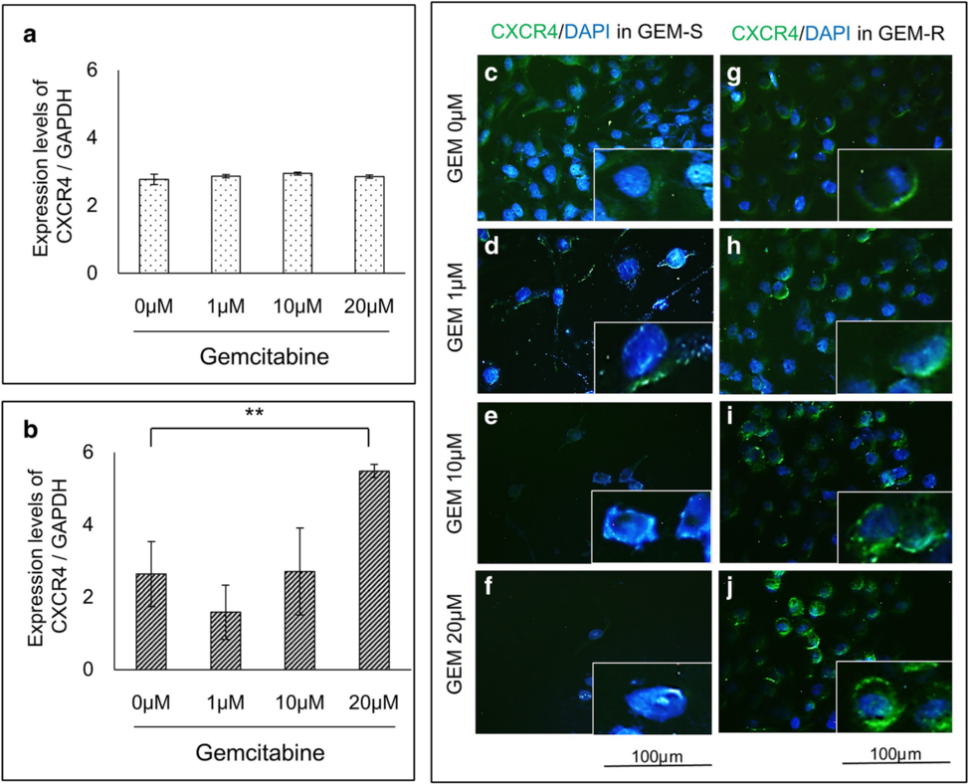
Alteration of *CXCR4* mRNA and protein expression in MIA PaCa-2 cells by GEM. PaCa cells were treated with different concentrations of GEM (0 - 20 μM) for 24 h. **a** The *CXCR4* mRNA levels in GEM-S and **b** in GEM-R were measured using RT-PCR (normalized to *GAPDH* expression). Values are expressed as means ± SD. Multiple comparisons were performed by using one-way ANOVA followed by Dunnett’s test. **, *P* < 0.01; *, *P* < 0.05 versus control (0 μM). **c-f** The expression of CXCR4 protein in GEM-S and **g-j** GEM-R was detected by immunostaining. CXCR4 (green) and DAPI (blue). Original magnification, ×200

The expression of CXCR4 protein by PaCa cells was examined by means of ELISA assays. In MIA PaCa-2, the expression of CXCR4 protein by GEM-S cells was significantly inhibited by GEM in a dose-dependent manner (*P* < 0.01) (Fig. 2a). In contrast, the expression of CXCR4 protein by GEM-R cells showed a significant dose-dependent enhancement by GEM (*P* < 0.01) (Fig. 2b). In AsPC-1, there was no change of expression of CXCR4 protein during GEM treatment of sensitive cells (Fig. 2c). However, the expression of CXCR4 protein by resistant cells significantly increased in the presence of GEM in a fashion that varied with the dose (*P* < 0.01) (Fig. 2d). Furthermore, in RT-PCR, there was no change of *CXCR4* mRNA levels by GEM treatment of sensitive MIA PaCa-2 cells (Fig. 3a). However, the level of *CXCR4* mRNA in GEM-R cells was significantly elevated by treatment with GEM in a dose-dependent manner (*P* < 0.01) (Fig. 3b).

In immunocytochemical assays, staining of CXCR4 protein was primarily found in the cell membrane of GEM-S and GEM-R cells. These cells were treated with GEM at concentrations of 0 μM, 1 μM, 10 μM and 20 μM. The staining of CXCR4 in GEM-R cells was enhanced by GEM as the dose was increased [CXCR4 (green) and DAPI (blue)] (Fig. 3c-j).

### The secretion of CXCL12 from FB was enhanced by co-culturing with GEM-R PaCa cells treated with GEM

**Fig. 4:**
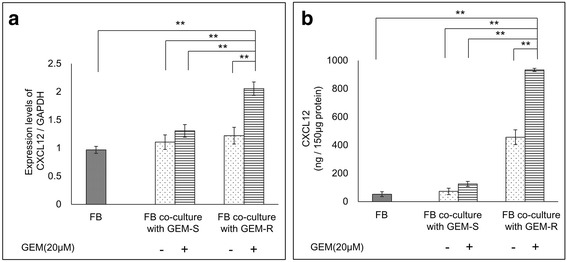
Alteration of *CXCL12* mRNA levels in fibroblasts (FB) resulting from co-culture with MIA PaCa-2 cells. FB were co-cultured for 24 h with GEM-R or GEM-S MIA PaCa-2 cells treated with or without GEM using a double-chamber method. **a** The mRNA levels of *CXCL12* in FB were measured using RT-PCR (normalized to *GAPDH* expression). Furthermore, after FB were co-cultured with PaCa cells for 72 h, the supernatants were collected from FB. **b** The concentrations of CXCL12 protein from FB were measured using an ELISA kit. Values are expressed as means ± SD. Multiple comparisons were performed using one-way ANOVA followed by the Bonferroni test. **, *P* < 0.01; *, *P* < 0.05

To investigate the function of the CXCL12-CXCR4 signaling axis, we determined whether the secretion of CXCL12 from FB was enhanced when FB were co-cultured with PaCa cells. The expression of *CXCL12* mRNA in FB was significantly enhanced by co-culturing with GEM-R PaCa cells treated with GEM (*P* < 0.01) (Fig.4a). Furthermore, the secretion levels of CXCL12 protein from FB were significantly enhanced by co-culturing with GEM-R PaCa cells treated with GEM (*P* < 0.01) (Fig. 4b).

### The role of CXCR4 in CXCL12-mediated invasiveness of GEM-R PaCa cells: inhibition by CXCR4 antagonists

**Fig. 5:**
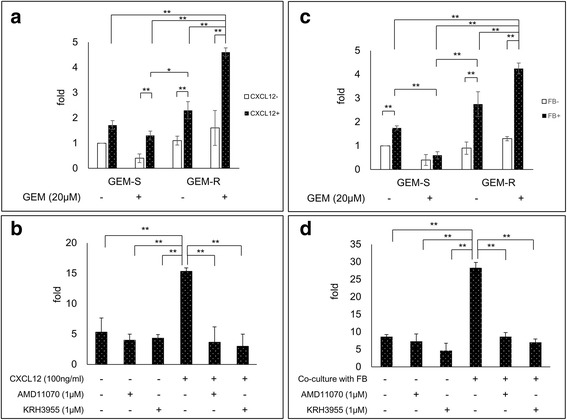
Alteration of invasiveness of PaCa cells by CXCL12 stimulation and by co-culture with FB. The invasiveness of GEM-R and GEM-S MIA PaCa-2 cells was assessed by a double-chamber method using a Matrigel invasion assay system. PaCa cells were seeded into Matrigel pre-coated Transwell chambers. These cells were allowed to migrate for 22 h. The cells that invaded through the membrane to the bottom of the upper chamber were fixed, stained, photographed and counted. The number of invading cells was counted in 5 random microscopic fields (200×). **a** The invasion assay was performed in basal medium containing recombinant CXCL12 (100 ng/mL) (**c**) and co-cultured with FB*.* **b** The effect of CXCR4 antagonists (AMD11070 and KRH3955) on the invasiveness of GEM-R PaCa cells treated with GEM activated by CXCL12 (**d**) and co-culturing with FB was examined. Values are expressed as means ± SDs. Multiple comparisons were performed by using one-way ANOVA followed by Bonferroni test. **, *P* < 0.01; *, *P* < 0.05

When GEM-R PaCa cells were exposed to GEM, the invasive behavior of these cells was significantly enhanced by stimulation with recombinant CXCL12 (*P* < 0.01) (Fig. 5a). Moreover, the enhanced invasive behavior of GEM-R PaCa cells was significantly inhibited by exposure to CXCR4 antagonists (*P* < 0.01) (Fig. 5b). Furthermore, when GEM-R PaCa cells were exposed to GEM, the invasive behavior of these cells was significantly elevated by co-culturing with FB (*P* < 0.01) (Fig. 5c). The activated invasive behavior of GEM-R PaCa cells was significantly inhibited by treatment with neutralizing CXCR4 antagonists (*P* < 0.01) (Fig. 5d). Photographs show alterations of invasive behavior of the PaCa cells in each treatment group (Additional file 1: Figure S1).

### GEM enhanced the growth of GEM-R PaCa cells in a nude mouse model: inhibition by CXCR4 antagonists

**Fig. 6:**
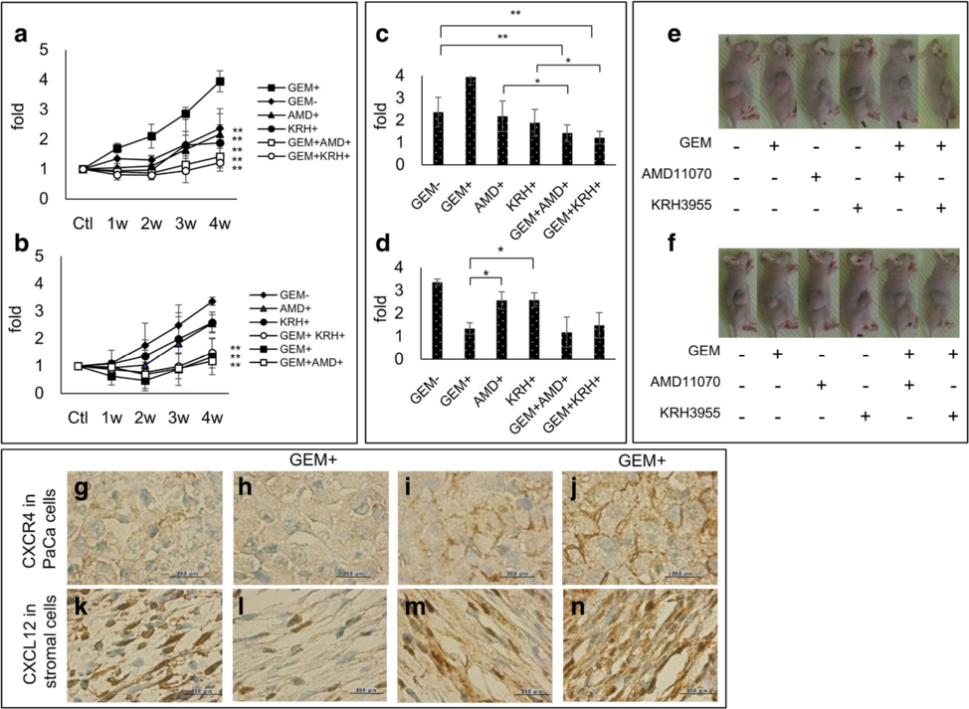
*In vivo* tumorigenicity of GEM-R PaCa cells and inhibition by CXCR4 antagonists*.* The growth of subcutaneous implanted GEM-S and GEM-R MIA PaCa-2 cells in nude mice. Mice were divided into 6 groups for each treatment: group I was not given any drugs, group II was given GEM, group III was given AMD11070, group IV was given KRH3955, groupV was given GEM and AMD11070 and groupVI was given GEM and KRH3955. The measurements of tumor volumes after implantation of **a** GEM-R or **b** GEM-S in each treatment group were plotted 4 weeks after beginning of the treatment. Values are expressed as means ± SD. Multiple comparisons were performed by using one-way ANOVA followed by Dunnett’s test, **, *P* < 0.01; *, *P* < 0.05 versus control (group II in GEM-R group, group I in GEM-S group at 4 weeks). The differences of tumor volumes after implantation of GEM-R (**c**) or GEM-S (**d**) were measured and photos showed representative results of GEM-R (**e**) and GEM-S (**f**) in each treatment group 4 weeks after beginning of the treatment. Values are expressed as means ± SD. Multiple comparisons were performed by using one-way ANOVA followed by the Bonferroni test, **, *P* < 0.01, *, *P* < 0.05 among all groups. Expression of CXCR4 protein determined by immunohistochemical staining (brown, CXCR4 protein, blue, nucleus) in implanted PaCa tumor: **g** GEM-S, **h** GEM-S treated with GEM, (**i**) GEM-R, (**j**) GEM-R treated with GEM. The secretion of CXCL12 from stromal cells around PaCa cells by immunohistochemical staining (brown, CXCL12 protein, blue, nucleus) in stromal cells, **k** stromal cells around GEM-S, **l** around GEM-S treated with GEM, **m** around GEM-R, **n** around GEM-R treated with GEM. Original magnification, ×1000, scale bars, 20 μm

On the basis of these results, we asked whether CXCR4 antagonists affected the growth of GEM-R PaCa cells *in vivo*, either alone or in combination with GEM. Six experimental conditions were tested (see Methods, Experimental Protocol). Using mice implanted with GEM-R 4 weeks earlier, the final tumor volume of group II (GEM+) was significantly greater than that found in any of the other groups (*P* < 0.01) (Fig. 6a). With GEM-S PaCa cells, the tumor volume of group I (no treatment) was significantly greater than groups II (GEM+), V (GEM+ AMD+) and VI (GEM+ KRH+) (*P* < 0.01) (Fig. 6b).

In GEM-R PaCa cells, tumor volumes in group V (GEM+ AMD+) and groupVI (GEM+ KRH+) were significantly less than groups III (AMD+) and IV (KRH+) (*P* < 0.05) and group I (no treatment) (*P* < 0.01) (Fig. 6c). There was no significant difference among groups I (no treatment), III (AMD+) and IV (KRH+). However, tumor volume in group II (GEM+) was significantly smaller than groups III (AMD+) and IV (KRH+) (*P* < 0.05) (Fig. 6d).

The photographs show the differences of the final tumor volume of all groups in GEM-R PaCa cells (Fig. 6e) and in GEM-S PaCa cells (Fig. 6f).

### Immunohistochemical analysis of *CXCR4* and *CXCL12* in implanted tumor tissue

CXCR4 protein was primarily identified in the cell membrane of PaCa cells. In contrast, it was not detected in normal stromal cells of noncancerous regions in PaCa tissue. Staining of CXCR4 protein in GEM-R cells treated with GEM was greatly enhanced (Fig. 6g-j). Significantly more CXCR4-positive cells were observed in GEM-R cells treated with GEM than GEM-R cells lacking such treatment (*P* < 0.05), GEM-S treated with GEM (*P* < 0.01) and GEM-S without GEM treatment (*P* < 0.01) (Additional file 2: Figure S2A). Staining of CXCL12 protein primarily occurred in the cytoplasm of stromal cells around PaCa cells, but it was not detected in PaCa tissues. Staining of CXCL12 protein was greatly enhanced in stromal cells around GEM-R treated with GEM (Fig. 6k-n). CXCL12 IOD values in stromal cells around GEM-R PaCa cells treated with GEM were significantly enhanced compared with other groups (*P* < 0.01) (Additional file 2: Figure S2B).

### The activity of NF-κB in GEM-R PaCa cells was enhanced by GEM

**Fig 7:**
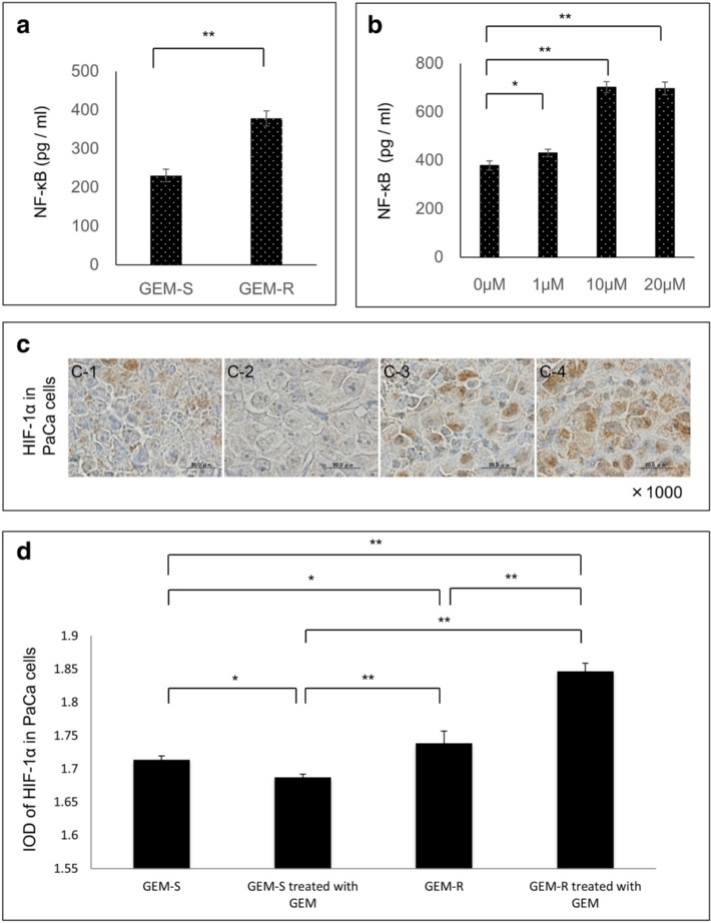
The expression of both NF-κB and HIF-1α in GEM-R and GEM-S PaCa cells. (**a**) The expression of NF-κB in GEM-R/S PaCa cells. The activity of NF-κB in GEM-R and GEM-S MIA PaCa-2 cells treated without GEM was measured by NF-κB (p65) transcription factor assay. Values are expressed as means ± SD. Between-group statistical significance was determined using the Student^’^s *t* test. **, *P* < 0.01. (**b**) The effect of GEM on NF-κB activity in GEM-R PaCa cells. GEM-R MIA PaCa-2 cells were treated with different concentrations of GEM (0–20 μM) for 72 h. The NF-κB p65 protein levels in GEM-R were measured. Values are expressed as means ± SD. Multiple comparisons were performed by using one-way ANOVA followed by Dunnett’s test. **, *P* < 0.01; *, *P* < 0.05 versus control (0 μM). (**c**) The expression of HIF-1α in implanted PaCa tumor. The expression of HIF-1α protein determined by immunohistochemical staining (brown, HIF-1α protein, blue, nucleus) in implanted PaCa tumor: (*c-1*) GEM-S, (*c-2*) GEM-S treated with GEM, (*c-3*) GEM-R, (*c-4*) GEM-R treated with GEM. Original magnification, ×1000, scale bars, 20 μm. (**d**) Quantification of immunostaining of HIF-1α protein by digital image analysis. For each image, the color deconvolution method was used to isolate HIF-1α-positive DAB-stained cells from HIF-1α-negative hematoxylin-stained cells. The measurement parameter was IOD. Optical density was calibrated and the area of interest was set as follows: hue, 0–30; saturation, 0–255; intensity, 0–255. The values were determined, and the IOD was log_10_ transformed. Values are expressed as means ± SD. Multiple comparisons were performed using one-way ANOVA followed by Bonferroni test, **, *P* < 0.01; *, *P* < 0.05

To examine the details of the molecular mechanisms, the activity of NF-κB in GEM-R/S PaCa cells was measured by NF-κB (p65) transcription factor assay. The activity of NF-κB in GEM-R MIA PaCa-2 cells was significantly higher compared to GEM-S MIA PaCa-2 cells (*P* < 0.01) (Fig. 7a). Moreover, the activity of NF-κB in GEM-R MIA PaCa-2 cells was significantly enhanced by GEM dose dependently (Fig. 7b).

### Immunohistochemical analysis of *HIF-1α* in implanted tumor tissue

Similarly, since HIF-1α might regulate the expression of CXCR4, we examined the expression of HIF-1α in implanted tumor tissue. Staining of HIF-1α protein in GEM-R cells treated with GEM was greatly enhanced (Fig. 7c). Significantly more HIF-1α-positive cells were observed in GEM-R cells treated with GEM than GEM-R cells lacking such treatment (*P* < 0.01), GEM-S treated with GEM (*P* < 0.01) and GEM-S without GEM treatment (*P* < 0.01) (Fig. 7d).

### The expression of CXCR7 mRNA in GEM-R PaCa cells

Since CXCR7 is another receptor of CXCL12, we examined the CXCR7 expression in GEM-R/S MIA PaCa-2 cells by RT-PCR. In RT-PCR, the expression levels of CXCR7 mRNA in GEM-R Mia PaCa-2 cells were significantly higher compared with GEM-S Mia PaCa-2 cells. There was no change of CXCR7 mRNA levels by GEM treatment of both GEM-R/S Mia PaCa cells (Additional file 3: Figure S3).

## Discussion

This study supports two conclusions. First, the resistance to GEM in PaCa cells was associated with activation of the CXCL12-CXCR4 signaling axis. Second, CXCR4 antagonists could inhibit the activation of the signaling axis and therefore restrain the invasive potency and tumorigenicity of GEM-R PaCa cells.

With regard to the first conclusion, the resistance to GEM in PaCa cells was associated with the activation of the CXCL12-CXCR4 signaling axis. Many different chemotherapeutic agents have failed to demonstrate any survival advantage in patients with PaCa. GEM has been the current standard of care for PaCa patients [3]; however, it has never proven to be very effective clinically for advanced PaCa cases because of the cells’ resistance to GEM. Improved therapeutic treatment will require a better understanding of the mechanisms by which these tumors become chemo-resistant and the development of strategies to overcome this resistance. Previous studies have suggested a variety of mechanisms of chemoresistance in PaCa, including the deregulation of key signaling pathways (such as NF-κB [28], phosphatidylinositol 3-kinase [PI3K]/Akt [29]), epithelial-mesenchymal transition (EMT) [30] and the presence of stromal cells [31]. In addition, a report suggested a relationship between CXCR4 and cancer stem cells. The report indicated that a subpopulation of migrating CD133+ CXCR4+ cancer stem cells was essential for tumor metastasis [32].

Recently, several studies detected the expression of CXCR4 and CXCL12 (also known as stromal-derived factor-1 [SDF-1]) in PaCa and stromal cells around PaCa cells. The CXCL12-CXCR4 signaling axis can promote PaCa tumorigenesis and chemoresistance in PaCa cells [17, 25, 26]. CXCR4, a member of the cell surface G-protein-coupled, seven-span transmembrane receptor family, is overexpressed in more than 20 types of human tumors, including breast cancer, prostate cancer, colorectal cancer, melanoma, neuroblastoma, and renal cell carcinoma [7]. High expression of CXCR4 is observed in the majority of PaCa tissues, and precancerous lesions play a role in PaCa pathogenesis [33]. Furthermore, high expression of CXCR4 correlates with poorer survival in PaCa patients after resection [34]. CXCL12, a ligand for CXCR4, is a chemokine that is constitutively secreted by several organs including lung, liver, small intestine, kidney, prostate, brain and skeletal muscle [35]. High amounts of CXCL12 are produced by organs commonly affected by cancer metastasis, such as lung and liver [14]. In CXCR4-positive PaCa cell lines, CXCL12 not only enhances chemotaxis, transendothelial migration and Matrigel invasion, but also stimulates cell proliferation and protects them from serum deprivation-induced apoptosis [36–38].

We focused on the relationship between the activated CXCL12-CXCR4 signaling axis and PaCa cells’ resistance to GEM. For that reason, we established two GEM-R PaCa cell lines. This is the first report to examine the importance of the CXCL12-CXCR4 signaling axis in resistance to GEM using GEM-R PaCa cells. Our study revealed that the expression of CXCR4 was significantly enhanced by GEM in GEM-R PaCa cells. Furthermore, when GEM-R PaCa cells were activated by GEM, they greatly increased the secretion of CXCL12 from FB. The invasiveness of GEM-R cells was also activated by CXCL12. *In vivo*, the tumorigenicity of GEM-R cells was enhanced by GEM. We confirmed that the CXCL12-CXCR4 signaling axis between tumor and stromal cells plays an important role in the invasiveness and tumorigenicity of GEM-R PaCa cells.

The second conclusion of this study was that CXCR4 antagonists could inhibit the activation of the signaling axis and could therefore restrain the invasiveness and tumorigenicity of GEM-R PaCa cells. CXCR4 antagonists were initially developed as new drugs for the treatment of HIV-1 infection. Among them, AMD3100, a specific antagonist of CXCR4, was initially considered to interfere with HIV-1 fusion through coating [18]. However, limitations of AMD3100 include a relatively short half-life (3.5 - 4.9 h) and the need to administer it via injection [39]. Furthermore, after long-term use, cardiotoxicity was noticed in patients [40, 41]. Due to those problems, clinical development was canceled. AMD11070 is a novel, orally bioavailable, selective, and reversible small-molecule antagonist of CXCR4 [19]. *In vitro*, it inhibits the binding of CXCL12 to CXCR4 and inhibits CXCL12–induced signaling mechanisms [42]. In two different ALL cells, equivalent concentrations of AMD11070 produced a stronger effect than AMD3100 [43]. Furthermore, no apparent acute toxicity was observed in oral bioavailability studies using AMD11070. The effectiveness of AMD11070 for malignant neoplasms was also reported in melanoma and lymphoblastic leukemia [43, 44]. Besides AMD11070, KRH3955 is also known as a CXCR4 antagonist. We decided to establish whether there was a similarity between the effect of KRH3955 and AMD11070. It was synthesized and purified by Kureha Corporation. KRH3955 showed oral bioavailability of 25.6 % in rats, and its oral administration blocked X4 HIV-1 replication in human peripheral blood lymphocytes and in a severely immunodeficient mouse system. The effect of KRH3955 on HIV was much higher than that of AMD3100 and AMD11070 [20]. However, there are no studies on the effect of KRH3955 on other malignant diseases.

We focused on the effects of CXCR4 antagonists, AMD11070 and KRH3955, on the invasiveness and tumorigenicity of GEM-R PaCa cells and determined whether these agents might represent a second line of chemotherapy for GEM-R PaCa cells. Our study revealed that when the invasiveness and tumorigenicity of GEM-R PaCa cells were activated by GEM, it was inhibited by the CXCR4 antagonists *in vitro* and *vivo*.

Previous report demonstrated that CXCR4 up-regulation by gemcitabine correlated with time-dependent accumulation of NF-κB and HIF-1α in the nucleus [45]. To examine the details of the molecular mechanisms, the activity of NF-κB in our GEM-R/S PaCa cells was measured by NF-κB (p65) transcription factor assay. The activity of NF-κB in GEM-R cells was significantly higher compared to GEM-S cells and was significantly enhanced by GEM dose dependently. Moreover, HIF-1α expression in GEM-R cells treated with GEM was greatly enhanced compared with GEM-R non-treated and GEM-S cells. So we will elucidate further molecular mechanisms of GEM-resistance in the next study.

CXCR7 is another receptor of CXCL12. Also, it is reported that CXCR7 plays important role in cancer invasion [46]. So besides CXCR4, we examined the expression of CXCR7 in GEM-R/S PaCa cells. The expression of CXCR7 in GEM-R PaCa cells is higher than GEM-S PaCa cells. So we may say that to some extent CXCR7 plays a role in GEM resistance. We are going to elucidate the details of the mechanisms of CXCR7 for GEM-resistance in the near future.

## Conclusion

In conclusion, we showed that GEM promoted the expression of CXCR4 in GEM-R PaCa cells and that activated GEM-R PaCa cells stimulated the secretion of CXCL12 from stromal cells. Finally, the CXCL12-CXCR4 signaling axis in GEM-R PaCa cells was activated by cooperative interactions between activated GEM-R PaCa cells and stromal cells, and this activation promoted GEM-R PaCa cell proliferation, invasion and tumorigenicity. Importantly, we have demonstrated that even when GEM-R PaCa cells were activated by GEM, the blockage of the *CXCL12-CXCR4* signaling axis by CXCR4 antagonists had impacts on GEM-R PaCa cell proliferation, invasion and tumorigenicity both *in vitro* and *in vivo.* Interestingly, these findings were observed only in GEM-R PaCa cells and not in GEM-S PaCa cells. As far as we know, this is the first study showing that one of several mechanisms of chemoresistance in PaCa cells involves chemokines and their receptor, *CXCL12*. We have shown that *CXCR4* antagonists can inhibit the development of GEM-R PaCa.

## Ethics approval and consent to participate

All animal studies were conducted in accordance with the guidelines established by the internal Institutional Animal Care and Use Committee and Ethics Committee guidelines of Nagoya City University.

## Consent for publication

Not applicable

## Availability of data and materials

The datasets supporting the conclusion of this article are included within the article and its additional files.

## Additional files

Additional file 1: Figure S1. Invasiveness of GEM-R and GEM-S PaCa cells and inhibition by CXCR4 antagonists. The photos showed alteration of invasiveness of PaCa cells by co-culturing with FB, and effect of CXCR4 antagonists, AMD11070 (1 μM) and KRH3955 (1 μM), on the invasiveness of PaCa cells. (JPG 874 kb) Additional file 2: Figure S2. Quantification of immunostaining of CXCR4 and CXCL12 protein by digital image analysis. (A) The number of CXCR4 immunoreactive cells in mouse specimens was expressed as a percentage of the total number of cells that were randomly counted in 10 fields at × 400 magnification. Furthermore, for each image, the color deconvolution method was used to isolate CXCL12-positive DAB-stained cells from CXCL12-negative hematoxylin-stained cells. The measurement parameter was IOD. Optical density was calibrated and the area of interest was set as follows: hue, 0–30; saturation, 0–255; intensity, 0–255. (B) The values were determined, and the IOD was log_10_ transformed. Values are expressed as means ± SD. Multiple comparisons were performed using one-way ANOVA followed by Bonferroni test, **, *P* < 0.01; *, *P* < 0.05. (JPG 295 kb) Additional file 3: Figure S3. Alteration of *CXCR7* mRNA expression in MIA PaCa-2 cells by GEM. PaCa cells were treated with different concentrations of GEM (0–20 μM) for 24 h. The expression of CXCR7 in GEM-R and GEM-S PaCa cells treated without GEM (A) was measured using RT-PCR (normalized to *GAPDH* expression). Values are expressed as means ± SD. Between-group statistical significance was determined using the Student^’^s *t* test. **, *P* < 0.01. The *CXCR7* mRNA levels in GEM-S (B) and in GEM-R (C) were measured using RT-PCR (normalized to *GAPDH* expression). Values are expressed as means ± SD. Multiple comparisons were performed by using one-way ANOVA followed by Dunnett’s test. **, *P* < 0.01; *, *P* < 0.05 versus control (0 μM). (JPG 374 kb)

## Abbreviations

PaCa: Pancreatic Cancer
GEM: Gemcitabine
GEM-R: Gemcitabine-Resistant
GEM-S: Gemcitabine-Sensitive
RT-PCR: reverse transcription polymerase chain reaction
ELISA: Enzyme Linked Immuno Solvent Assay
FB: Fibroblast cells
EMT: epithelial-mesenchymal transition
SDF-1: stromal-derived factor-1
DMEM: Dulbecco’s Modified Eagle’s Medium
RPMI-1640: Roswell Park Memorial Institute-1640
FBS: fetal bovine serum
IC50: the half maximal inhibitory concentration
DPEC: diethylpyrocarbonate
PBS: Phosphate Buffered Saline
DAB: 3,3-diaminobenzidine tetrahydrochloride
RGB: red-green-blue
IOD: integrated optical density
SD: standard deviation
NF-κB: Nuclear factor-kappa B
HIF-1α: Hypoxia-Inducible Factor-1α
