# Supplementary figures and images for: Enhancement of the CXCL12/CXCR4 axis due to acquisition of gemcitabine resistance in pancreatic cancer: effect of CXCR4 antagonists
Source: BMC Cancer. 2016 May 12;16:305. doi: 10.1186/s12885-016-2340-z (PMC4866076; doi:10.1186/s12885-016-2340-z)

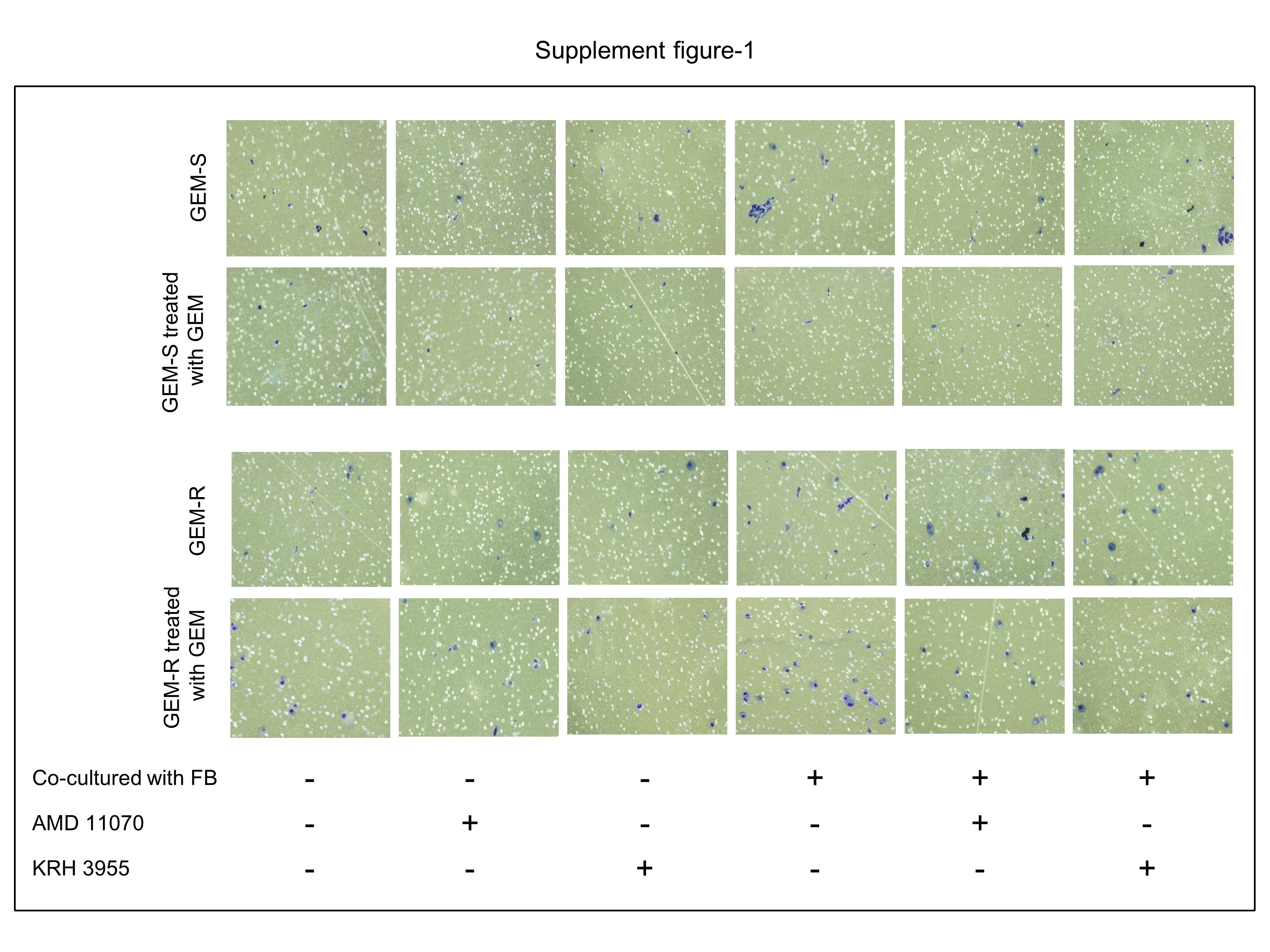

Supplement: Additional file 1: Figure S1. — Invasiveness of GEM-R and GEM-S PaCa cells and inhibition by CXCR4 antagonists. The photos showed alteration of invasiveness of PaCa cells by co-culturing with FB, and effect of CXCR4 antagonists, AMD11070 (1 μM) and KRH3955 (1 μM), on the invasiveness of PaCa cells. (JPG 874 kb) [file 12885_2016_2340_MOESM1_ESM.jpg]

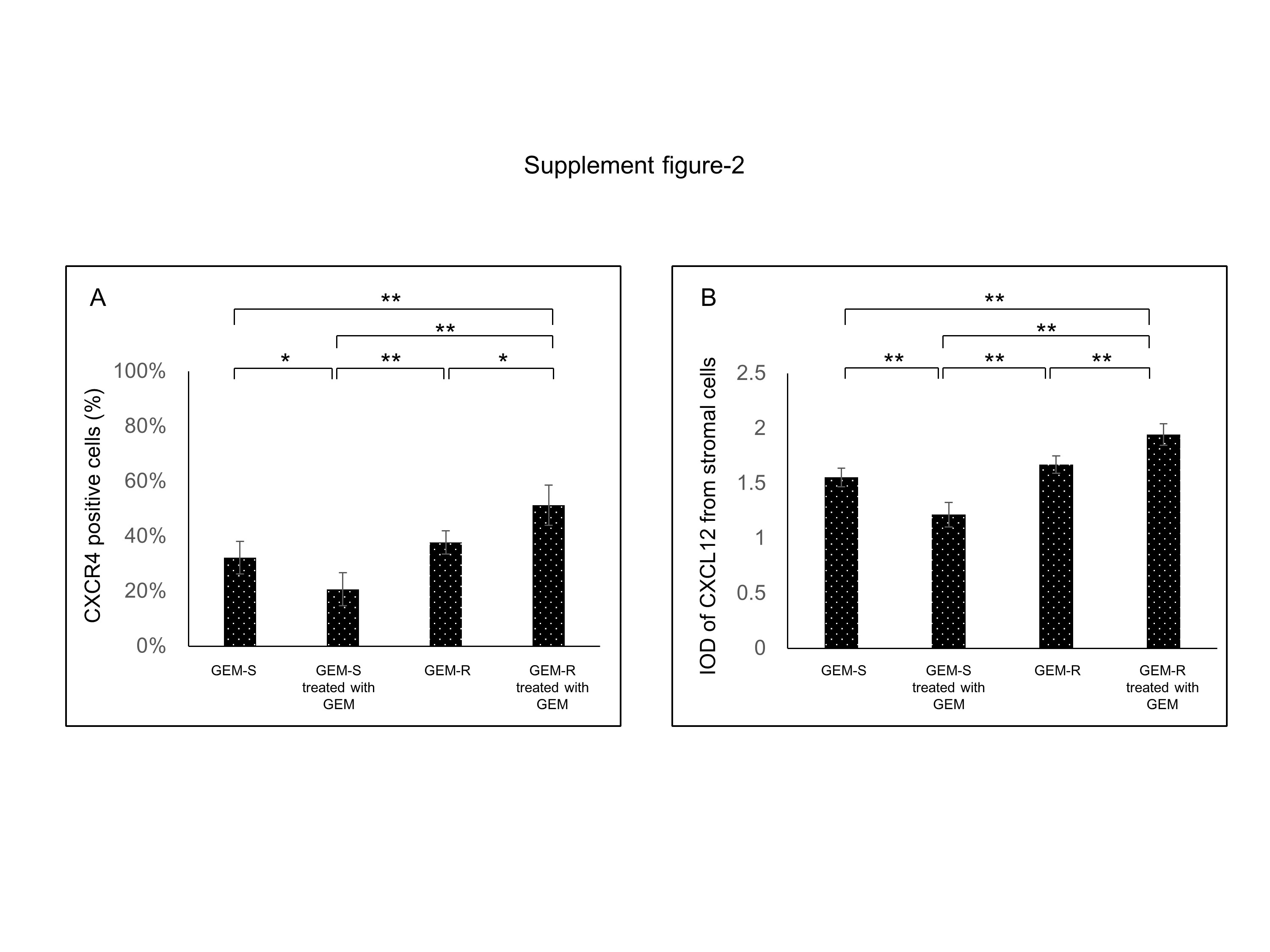

Supplement: Additional file 2: Figure S2. — Quantification of immunostaining of CXCR4 and CXCL12 protein by digital image analysis. (A) The number of CXCR4 immunoreactive cells in mouse specimens was expressed as a percentage of the total number of cells that were randomly counted in 10 fields at × 400 magnification. Furthermore, for each image, the color deconvolution method was used to isolate CXCL12-positive DAB-stained cells from CXCL12-negative hematoxylin-stained cells. The measurement parameter was IOD. Optical density was calibrated and the area of interest was set as follows: hue, 0–30; saturation, 0–255; intensity, 0–255. (B) The values were determined, and the IOD was log10 transformed. Values are expressed as means ± SD. Multiple comparisons were performed using one-way ANOVA followed by Bonferroni test, **, P < 0.01; *, P < 0.05. (JPG 295 kb) [file 12885_2016_2340_MOESM2_ESM.jpg]

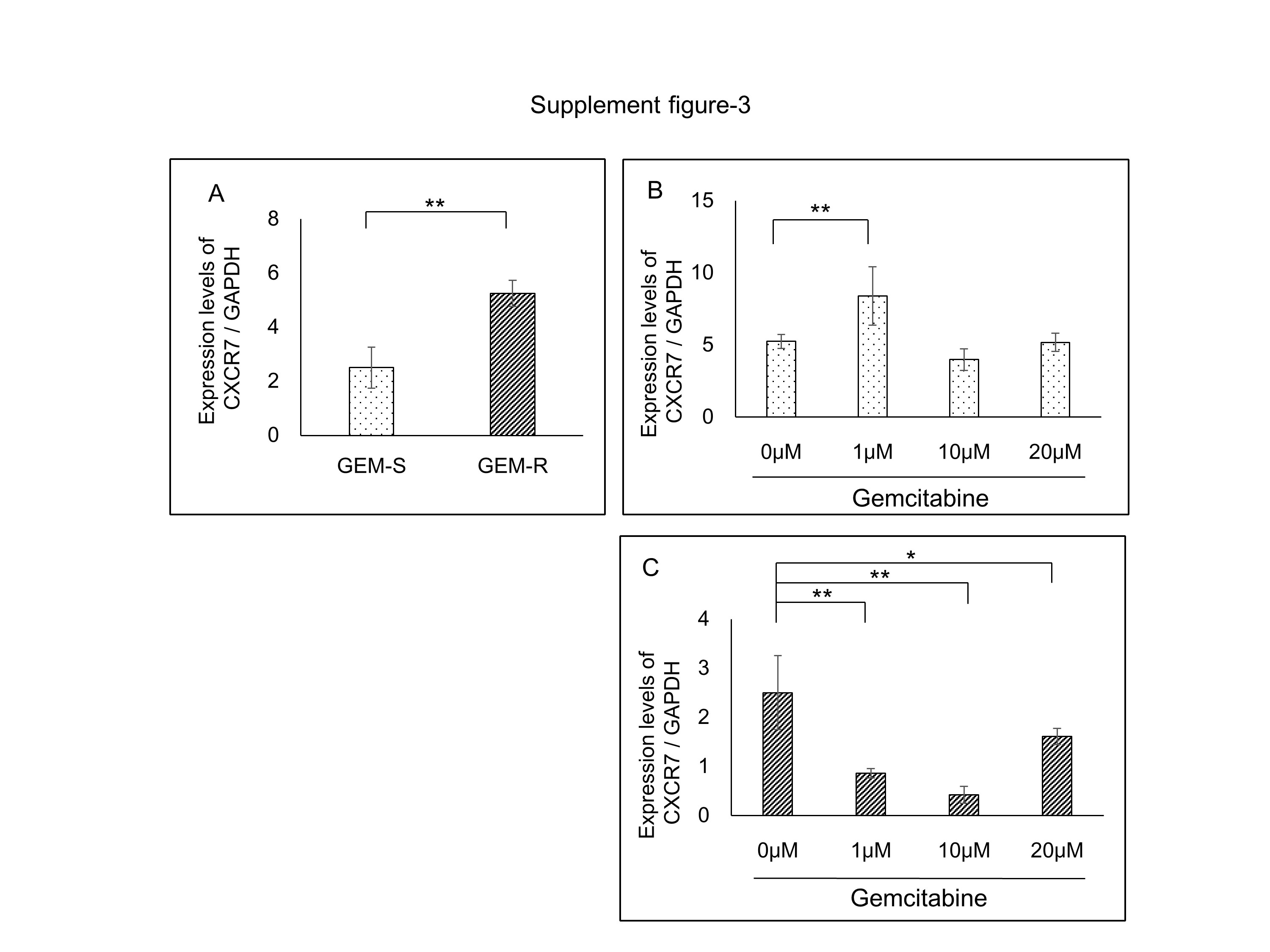

Supplement: Additional file 3: Figure S3. — Alteration of CXCR7 mRNA expression in MIA PaCa-2 cells by GEM. PaCa cells were treated with different concentrations of GEM (0–20 μM) for 24 h. The expression of CXCR7 in GEM-R and GEM-S PaCa cells treated without GEM (A) was measured using RT-PCR (normalized to GAPDH expression). Values are expressed as means ± SD. Between-group statistical significance was determined using the Student’s t test. **, P < 0.01. The CXCR7 mRNA levels in GEM-S (B) and in GEM-R (C) were measured using RT-PCR (normalized to GAPDH expression). Values are expressed as means ± SD. Multiple comparisons were performed by using one-way ANOVA followed by Dunnett’s test. **, P < 0.01; *, P < 0.05 versus control (0 μM). (JPG 374 kb) [file 12885_2016_2340_MOESM3_ESM.jpg]
